# Supplementary material for: Engineering high quality graphene superlattices via ion milled ultra-thin etching masks
Source: Nat Commun. 2022 Nov 14;13:6926. doi: 10.1038/s41467-022-34734-3 (PMC9663573; doi:10.1038/s41467-022-34734-3)
Supplement: Supplementary file 1 — Supplementary information [file 41467_2022_34734_MOESM1_ESM.pdf]

Supplementary Information for:

Engineering high quality graphene superlattices via ion milled ultra-thin etching masks

Authors: David Barcons Ruiz[1], Hanan Herzig Sheinfux[1], Rebecca Hoffmann[1], Iacopo Torre[1], Hitesh Agarwal[1], Roshan Krishna Kumar[1], Lorenzo Vistoli[1], Takashi Taniguchi[2,3], Kenji Watanabe[2,3], Adrian Bachtold[1,4], Frank H.L. Koppens*[1,4]

[1] ICFO - Institut de Ciencies Fotoniques, The Barcelona Institute of Science and Technology, 08860 Castelldefels (Barcelona), Spain
[2] Research Center for Functional Materials, National Institute for Materials Science, Tsukuba, Japan
[3] International Center for Materials Nanoarchitectonics, National Institute for Materials Science, Tsukuba, Japan
[4] ICREA - Institució Catalana de Recerca i Estudis Avançats, 08010 Barcelona, Spain

Corresponding author: *frank.koppens@icfo.eu

Supplementary Note 1. Details of the graphite gate patterning

In Supplementary Figure 1 we depict the transfer process of the silicon membrane and the etching of the graphite flake in detail. Moreover, we show in Supplementary Figure 2 optical and AFM images of every step in the fabrication process of a 30 nm period hexagonal lattice.

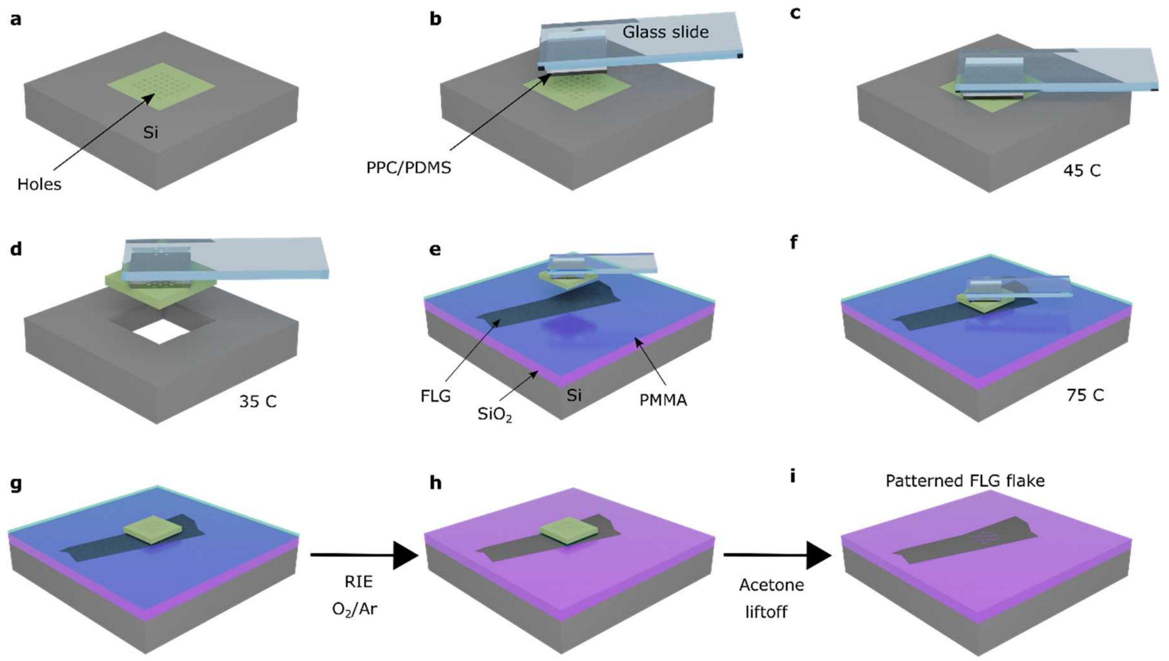

Supplementary Figure 1. The fabrication process of a patterned graphite gate. a. A suspended silicon membrane, as thin as 5 nm, is milled with FIB. **b-d.** The membrane is detached and picked up from its supporting frame with a polymeric stamp made of PPC/PDMS[1]. **e.** A Si/SiO_2 substrate with a few-layer graphene flake (FLG) is prepared in parallel, with a very thin layer of PMMA spin coated on the surface. **f,g.** The milled membrane is released on the new substrate by heating up to decrease the adhesion to the PPC film. **h.** A standard O_2/Ar RIE process is followed to transfer the pattern from the membrane to the FLG. **i.** Finally, the sample is sonicated in acetone for a short time to clean the PMMA and remove the silicon membrane.

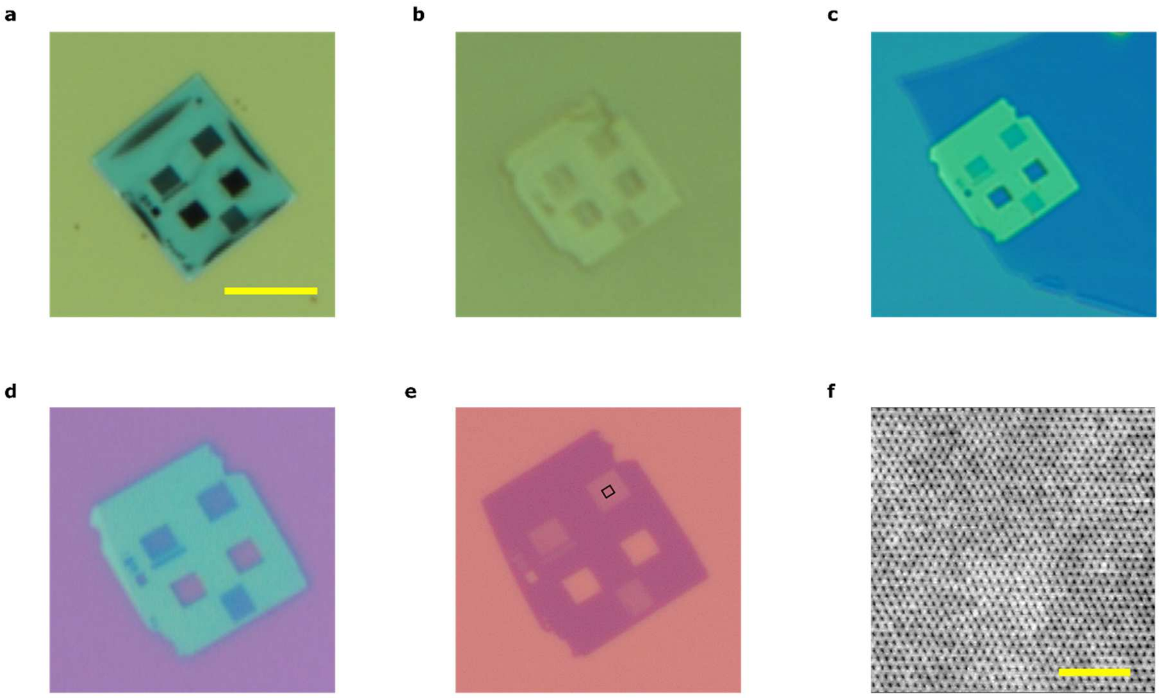

Supplementary Figure 2. Fabrication of a 30 nm period hexagonal lattice on a FLG. a. A 15x15 um^2 and 10 nm thin Si membrane is patterned with a He FIB. The scale bar is 10 µm. **b.** The membrane is picked up with a PPC/PDMS stamp and **c**. dropped on the target FLG flake (around 3 nm thick). **d.** An O_2/Ar RIE process is followed (20W, O_2/Ar, 40/40 sccm, 10 Pa, 1’40”) and **e.** finally the silicon membrane is removed by acetone sonication for 2 minutes, followed by 15 minutes of acetone cleaning plus 3 hours vacuum annealing at 600 C. **f.** AFM topography image of the resulting 30 nm hexagonal lattice. The scale bar is 250 nm.

Supplementary Note 2. Limitations of the technique

There are some limitations that compromise the resolution limit when patterning highly dense lattices

- One of the main limitations is the mechanical stability of the suspended mask. Due to its nanometric thickness, milling a highly dense lattice where a big fraction of the material is removed results in holes merging or big areas breaking due to stress relaxation. In particular, producing patterns with very fine periods and large overall areas is further complicated by any minimal amount of drift or deformation in the long range order of the milled pattern, which cause neighboring holes (or hole rows) to overlap.
These issues can largely be alleviated by using alternate custom-made membrane windows with thinner membrane thickness or by using mechanically stronger materials (e.g. non stochiometric SiN). Thinning the membrane contributes to reducing the milling dose, thereby reducing secondary ion collision damage and allowing the usage of higher resolution (lower current) HIM beam conditions.

- Related to the mechanical stability of the membrane is the aspect ratio of the patterned membrane – the ratio of the hole size (lattice period) to the membrane thickness. While He FIB milling is known to achieve high aspect ratios, we have observed that increasing the membrane thickness decreases the achievable resolution. This is most likely due to variations in the hole size between the top and bottom of the membrane (in addition to the increased aforementioned secondary ion collision damage).

- It is therefore expected that using dedicated Si membranes with thinner Si windows will enable further reduction in the minimal lattice period. Similarly, alternate membrane window materials can be used, which are mechanically stronger than polycrystalline Si (e.g. ultrathin nonstoichiometric SiN). In this regard, it should be noted that while the membranes used are nominally 5 nm thick, the actual thickness measured in AFM (after transfer to substrate) is larger, on the order of $10\ \mathrm{nm}$. Importantly, the membrane thickness (and the milling dose) does not change appreciably between different membranes (including those produced in different fabrication batches).

- Another part of the process which suffers from limited mechanical stability is the transfer process to the target substrate, where the membrane is picked up (broken) from the window frame and attached to a polymer. Due to the adherence properties of PPC, we change the temperature between the different steps in the transfer process. Even though the mask survives the transfer, we sometime observe long range disorder appearing in highly dense lattices due to stretching/compression of the mask. We attribute this to the thermal expansion of PPC. We expect that further optimization of the transfer process may reduce the number of such disordered lattices.

- As explained in the previous section, a buffer layer on the target substrate is needed to allow for a reliable lift off after etching. This results in a higher aspect ratio for the etching process, limiting the minimum feature size. It is conceptually possible to avoid using the buffer PMMA layer and remove the membrane by etching. We made experiments using SF_6 plasma for etching, but found the process less reliable then the PMMA based liftoff technique, due to the very sensitive optimization needed to remove the Si mask completely without fluorinating and partially etching the graphite flake.

Supplementary Note 3. Heterostructures and device's micrographs

We provide optical micrographs before patterning the hall bars and after, for Dev 1 (Supplementary Figure 3) and Dev 2 (Supplementary Figure 4).

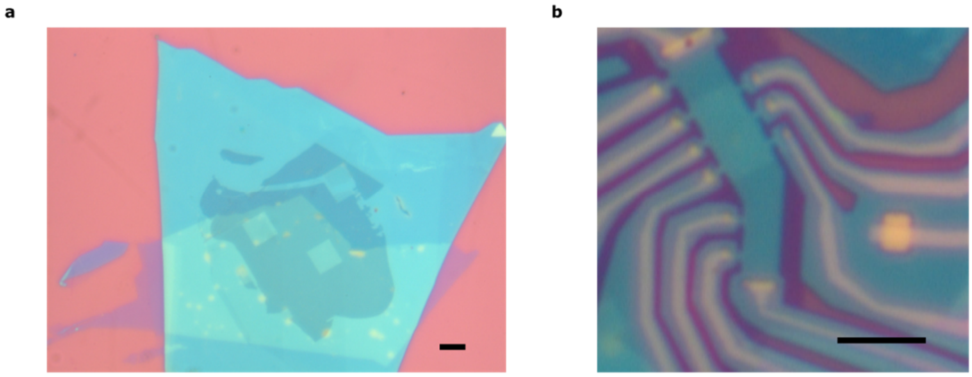

Supplementary Figure 3. a. Optical micrograph of device 1 before patterning the hall bar. **b.** Same as (a), but after patterning. The scale bar is 5 µm in both cases.

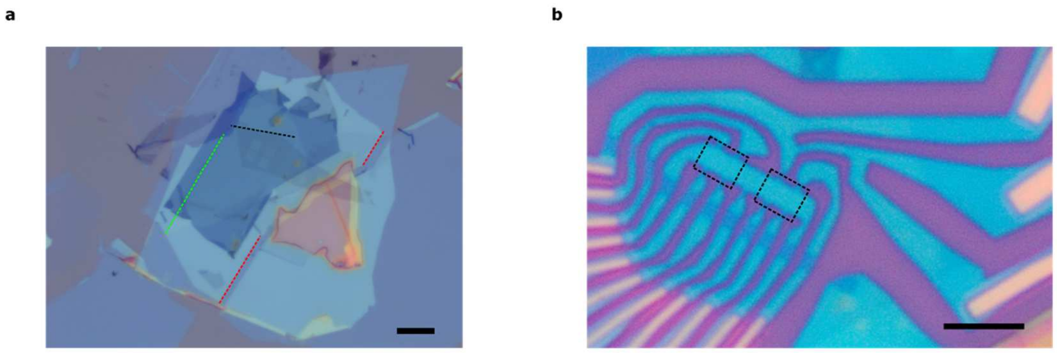

Supplementary Figure 4. a. Optical micrograph of device 2 before patterning the hall bar. The red and green dashed lines indicate the crystal axes of the top and bottom hBN flakes, respectively. The black dashed line indicates a graphene pristine edge, which determines its crystallographic orientation. Therefore, we discard hBN alignment in this sample. **b.** Optical micrograph of the same device after fabricating the hall bar. Black dashed regions indicate the patterned superlattice regions. The scale bar is 5 µm in both cases.

Supplementary Note 4. Electrostatic potential profile calculation

We estimated the electronic-density profile induced in graphene by the patterned gate by solving the equation of electrostatic using the Finite Element Method. For the sake of simplicity, we performed 2D simulations in a 1D-periodic geometry with the same lateral dimension of the real devices. We do not expect the solution of the full 3D equation to change the results qualitatively.

We considered the non-linearity due to the quantum capacitance of graphene, and we used the following values of the dielectric constants of SiO_2 ($\varepsilon = 3.9$) and hBN ($\varepsilon_{zz} = 3.56$, $\varepsilon_{xx} = 6.7$). We implemented the FEM using a Python code based on the open-source library FEniCS[2].

In Supplementary Figure 5 we show a side cut of Dev 1 and Dev 2, and their calculated electrostatic doping profile for a line cut crossing one hole. The voltage settings used are those that produce an average density $n/n_0 = -4$.

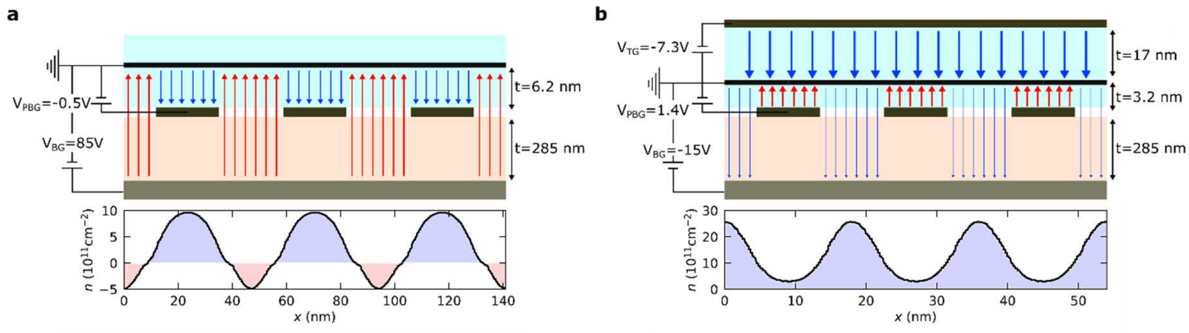

Supplementary Figure 5. a,b. Induced carrier density modulation for $n/n_0 = -4$ voltage configuration for Dev 1 and Dev2, respectively. In both cases, the diameter of the holes is estimated to be $0.5 \cdot a_{SL}$ from the AFM topography images. The gate voltages chosen are those used for the magnetoresistance measurements.

Supplementary Note 5. Band structure and conductivity calculation

We solve the following Hamiltonian for graphene under an external periodic scalar potential $U(\boldsymbol{r})$ in two dimensions

$$\boldsymbol{\mathcal{H}} = v_D \boldsymbol{\sigma} \cdot \boldsymbol{p} + U(\boldsymbol{r})\mathbb{I} \, .$$

The scalar potential $U(\boldsymbol{r})$ is chosen to be a muffin-tin potential

$$U(\boldsymbol{r}) = u \sum_{\boldsymbol{t}} f_{MT}(|\boldsymbol{r} - \boldsymbol{t}|) \, ,$$

with u being the potential amplitude and $f_{MT}(|\boldsymbol{r}|)$ representing a smooth potential well of radius r_0 and smoothing length s. The potential is expanded in a Fourier series with the reciprocal lattice vectors of the superlattice $\boldsymbol{G} = N_1\boldsymbol{g_1} + N_2\boldsymbol{g_2}$

$$U(\boldsymbol{r}) = \sum_{\boldsymbol{G}} U_{\boldsymbol{G}} e^{i\boldsymbol{G}\cdot\boldsymbol{r}} \, ,$$

with

$$U_{\boldsymbol{G}} = \begin{cases} u \frac{\tilde{f}_{MT}(G, r_0, s)}{\Omega_{u.c.}} & if \ \boldsymbol{G} \neq \boldsymbol{0} \\ 0 & if \ \boldsymbol{G} = \boldsymbol{0} \end{cases} .$$

Here, $\tilde{f}_{MT}(G, r_0, s) = \frac{2\pi r_0}{G} J_1(r_0 \, G) e^{-G^2 s^2}$ the Fourier transform of the well potential. We include enough $\boldsymbol{G}$ vectors such that $\hbar v_D G_{max} \gg \Delta U_{max}$

By adjusting the patterned hole radius r_0 and the smoothing parameter s, we are able to reproduce the potential profile calculated in the previous section.

In Supplementary Figure 6 we plot the calculated band structure for both devices discussed in the main paper, and the inverse Drude weight, which is proportional to the band conductivity.

Importantly, the effective potential modulation induced in the graphene is also influenced by in-plane screening, to a degree that depends on the lattice period. Furthermore, the typical dependence of screening in monolayer graphene on the carrier density means that the experienced modulation will be smaller for the higher order Dirac peaks, which in general can have a bearing on interpreting experimental results.

To account for the in-plane screening, we consider the random phase approximation (RPA), similar to its application in [3]. The effective magnitude of the potential in the graphene layer, u_{eff} is reduced by a factor of $1/\epsilon$, where

$$\epsilon = 1 - v_{G_0} \tilde{\chi}(G_0; \omega = 0),$$

with $G_0 = \pi / a_{SL}$, $\tilde{\chi}(G_0; \omega = 0)$ being the d.c. susceptibility of monolayer graphene and v_q the Coulomb interaction potential

$$v_q = \frac{2\pi e^2}{q\bar{\epsilon}} \left[1 - \frac{e^{-2q\eta t_1} - 2e^{-2q\eta(t_1+t_2)} + e^{-2q\eta t_2}}{1 - e^{-2q\eta(t_1+t_2)}} \right],$$

with t_1, t_2 being the thicknesses of the top and bottom hBN spacers ($t_1 \to \infty$ for Dev 1 since there is no top gate) and $\bar{\epsilon} \approx 4.88, \eta \approx 1.37$ being the hBN permittivity parameters.

Supplementary Figure 7 shows the screening effect in our devices, where the potential reduction is slightly stronger for the shorter period device, Dev 2, than for Dev 1. The effect of the screening depends on the SL filling fraction, i.e. carrier density. However, the variation is relatively slow. For

example, the induced potential does not change dramatically (in the RPA) between filling fractions 4 and 8.

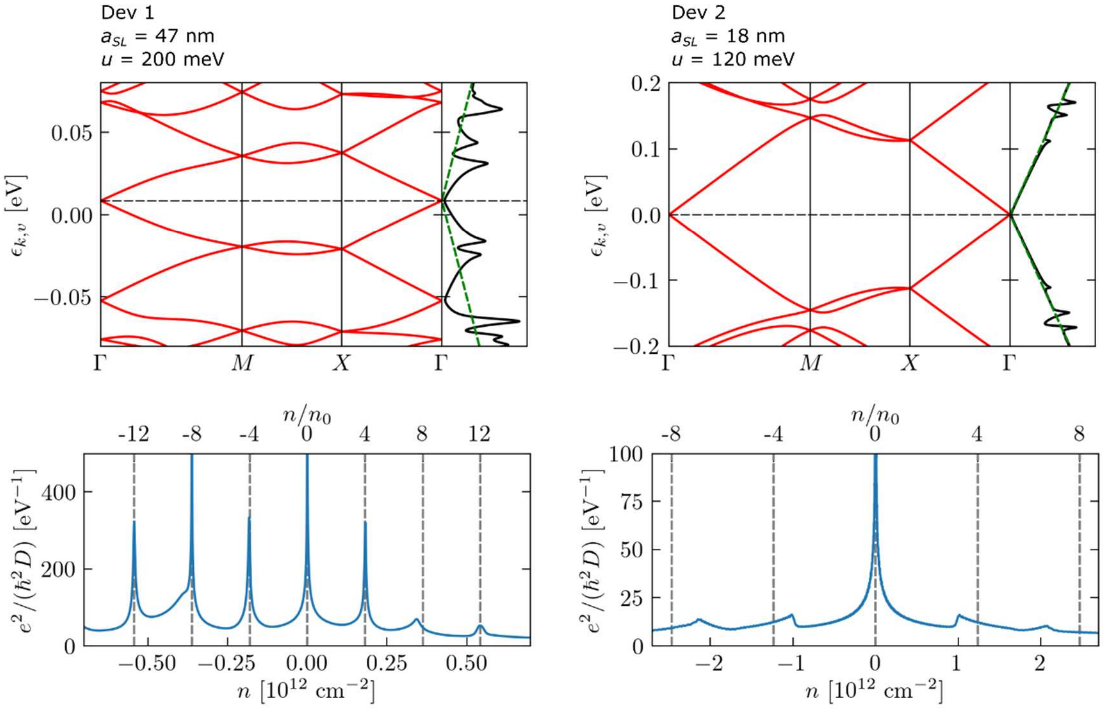

Supplementary Figure 6. Calculated band structure, density of states and inverse of Drude weight for Dev 1 and 2. We consider the potential modulation for the calculation obtained from our electrostatics model. In the top panels, we plot the bands along the high symmetry point in the BZ of a square lattice, together with the density of states for the case with the modulation (black line) and the zero-modulation case (green dashed line). Bottom panels display the inverse of the Drude weight as a function of carrier density and filling fraction of the SL n/n_0.

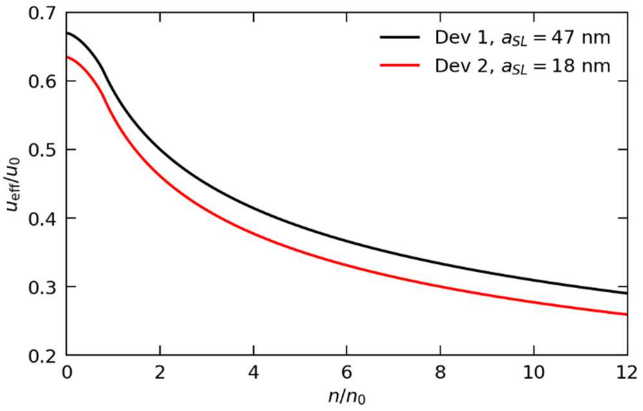

Supplementary Figure 7. Effective electrostatic potential reduction u_{eff}/u_0 due to in-plane screening in the RPA, for Dev 1 and 2, as a function of the SL filling fraction n/n_0.

Supplementary Note 6. Longitudinal resistivity map for Dev 1

In Supplementary Figure 8 we show the emergence of the satellite peaks as a function of Si BG. The peaks appear as parallel lines to the main Dirac peak, and scale up in resistance as both gates are increased, i.e. towards higher modulation.

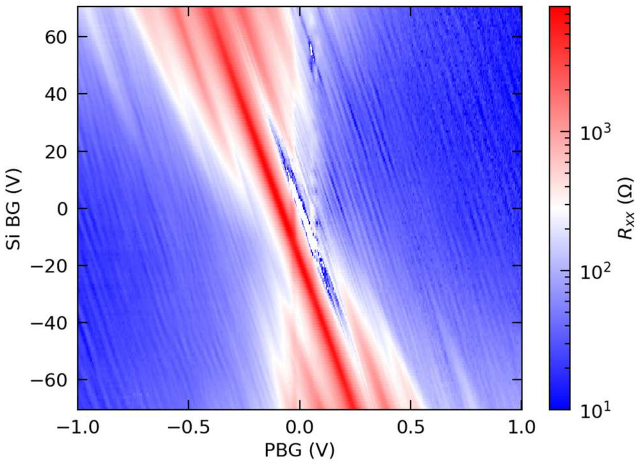

Supplementary Figure 8. Dev 1 longitudinal resistance as a function of patterned gate voltage (PBG) and silicon backgate (Si BG). Parallel resistance lines to the main one are the so called satellite peaks, which occur for multiples of $4n/n_0$.

Supplementary Note 7. Extra data for Dev 2

In Fig. 3e we show hall effect measurements for Dev 1. In Supplementary Figure 9 we show a similar measurement (hall effect and longitudinal resistivity) for Dev 2 for two values of the PBG.

In Supplementary Figure 10, we display as well the relative change in resistivity respect to the $V_{PBG} =$ 0 V case. This figure shows with more clarity the effect of the patterned gate on Dev 2.

In generating the plot in Fig. 3f of the main text, as well the plot above, it should be noted that we have manually removed parts of the plot where a measurement artifact appears, which obscures the measured signal. This artifact is specific to the measurement configuration and not to the patterned region of our device. In a TG-PBG voltage resistance map of our device (Supplementary Figure 11), this artifact appears as a diagonal line with different slope to our main Dirac peak line. The different slope means a different capacitance, indicating that this line is a measurement artifact not related to our patterned region.

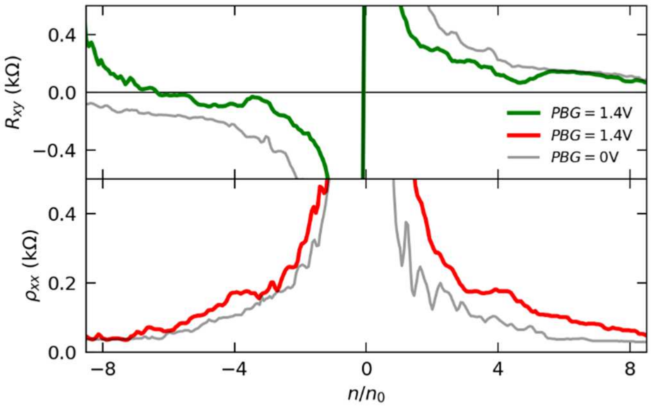

Supplementary Figure 9. Longitudinal resistivity (red trace) and Hall resistance at $B = 0.2$ T (green trace) as a function of the electron density per SL unit cell for Dev 2 at $V_{PBG} =$ 1.4 V. Grey traces display longitudinal resistivity and Hall resistance for $V_{PBG} =$ 0 V (Dev 2).

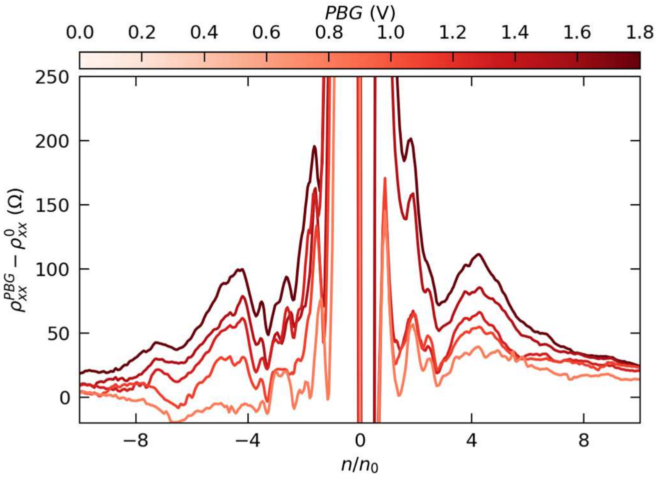

Supplementary Figure 10. Excess resistivity with respect to the $V_{PBG} = 0$ V case for Dev 2. Up to 100 Ω excess resistivity is observed at $n/n_0 = \pm 4$, while it drops at higher carrier densities. Further experiments are needed to verify and reproduce the excess resistivity peak at $n/n_0 = \pm 2$, meaning the presence of correlations.

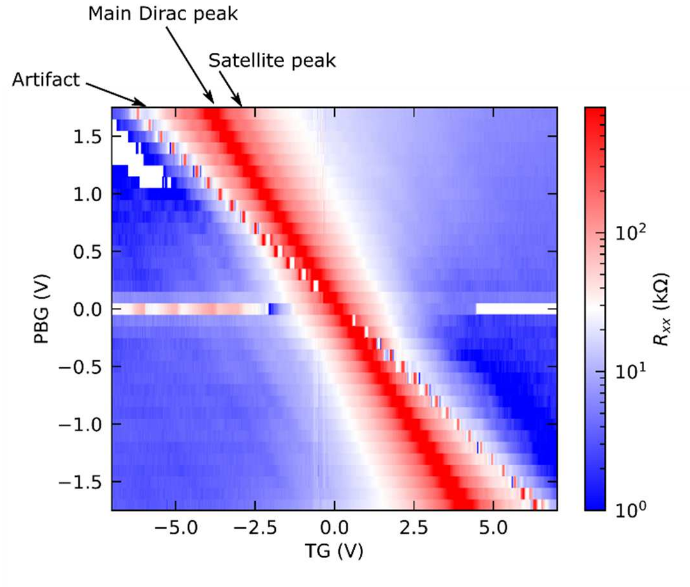

Supplementary Figure 11. Dev 2 longitudinal resistance as a function of patterned gate voltage (PBG) and top gate voltage (TG). We indicate with arrows the origin of the different resistance lines.

Supplementary Note 8. AFM image filtering

Since AFM topography images suffer from substrate (SiO_2) height variations at the sub nanometer scale, we apply a high pass filter with cut off frequency $f = 1/3 \cdot f_{a_{SL}}$ by performing a 2D FFT. In Supplementary Figure 12 we show the AFM image from Fig. 2 before and after processing.

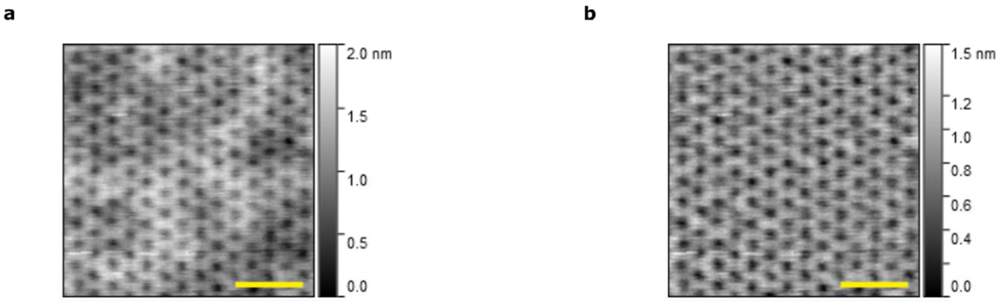

Supplementary Figure 12. a. Unprocessed AFM topography image of a triangular 16 nm lattice patterned on a graphite flake on a Si/ SiO_2 substrate. **b.** Image in a but processed with a high pass filter. The scale bar is 50 nm.

Supplementary References

1. Pizzocchero, F. *et al.* The hot pick-up technique for batch assembly of van der Waals heterostructures. *Nat. Commun.* **7**, 11894 (2016).

2. Alnaes, M. S. *et al.* The FEniCS Project Version 1.5. *Arch. Numer. Softw.* **3**, 9–23 (2015).

3. Wunsch, B., Stauber, T., Sols, F. & Guinea, F. Dynamical polarization of graphene at finite doping. *New J. Phys.* **8**, 318–318 (2006).
